# Selective Autophagy Mediated by Protein Ubiquitination in Major Prevalent Zoonoses

**DOI:** 10.1155/tbed/6238787

**Published:** 2025-03-22

**Authors:** Chi Meng, Fengyuan Jiao, Gengxu Zhou, Lingjie Wang, Shengping Wu, Cailiang Fan, Jixiang Li, Liting Cao, Zuoyong Zhou, Yuefeng Chu, Hanwei Jiao

**Affiliations:** ^1^ The College of Veterinary Medicine, Southwest University, Chongqing, 402460, China, southwest.edu; ^2^ Animal Epidemic Prevention and Control Center of Rongchang, Chongqing, 402460, China; ^3^ State Key Laboratory for Animal Disease Control and Prevention, College of Veterinary Medicine, Lanzhou University, Lanzhou Veterinary Research Institute, Chinese Academy of Agricultural Sciences, Lanzhou, Gansu, 730000, China, caas.cn

**Keywords:** regulatory mechanisms, selective autophagy, ubiquitination modification, zoonotic diseases

## Abstract

Zoonotic diseases not only cause great harm to animal health but also involve the development of animal husbandry, which in turn endangers human life and health and public health safety. Protein ubiquitination and autophagy are important ways for the body to degrade invading pathogens, which correspond to the ubiquitin (Ub)‐proteasome system (UPS) and autophagic lysosomal pathway (ALP), respectively, and play an important role in the occurrence and development of diseases. For UPS, the substrate is delivered to the 26S proteasome system via a ubiquitination cascade and subsequently degraded and removed. For ALP, the substrate is encapsulated to form autophagosomes, which subsequently fuse with lysosomes to form autophagolysosomes, which are eventually degraded and cleared. However, a variety of zoonotic pathogens can interfere with the protein ubiquitination pathway and autophagy process to promote self‐replication and survival, and resist host immune defense. This article reviews the mechanisms by which multiple pathogens interfere with protein degradation pathways, providing a new perspective for the treatment and prevention of zoonotic diseases.

## 1. Introduction

Animal husbandry production is closely related to human activities, and the occurrence of zoonotic diseases seriously threatens the public health and safety of society. Zoonotic diseases are diseases that are naturally transmitted from vertebrates to humans and are transmissible across species and in a wide range of ways, either through direct contact or through food, water, or the environment. The causative agents of zoonotic diseases can be bacteria, viruses, parasites, etc. [1]. The ubiquitin (Ub)‐proteasome system (UPS) and the autophagic lysosomal pathway (ALP) are the two major pathways for protein degradation in cells, which are essential for resisting and removing pathogens and maintaining cellular homeostasis.

As an important protein posttranslational modification, ubiquitination is involved in protein degradation and quality control, endocytosis, vesicle trafficking, cell cycle control, stress response, DNA repair, growth factor signaling, and other life activities [2, 3]. The Ub system is involved in regulating intracellular signal transduction, maintaining cellular homeostasis, responding to cellular stress damage, participating in autoimmune diseases, inflammatory responses, autophagy, etc. [4]. Autophagy is a lysosome‐dependent self‐degradation system that is essential for maintaining cellular homeostasis and energy balance [5]. Selective autophagy plays a key role in the removal of misfolded or aggregated proteins, the removal of damaged organelles such as mitochondria, endoplasmic reticulum, and peroxisomes, and the elimination of intracellular pathogens [6]. In addition, dysregulation of autophagy has been associated with a variety of diseases, including cancer [7], tumors [8], and neurodegenerative diseases [9].

## 2. Ubiquitination Modification and Selective Autophagy

### 2.1. Ubiquitination Modification and UPS

**Figure 1:**
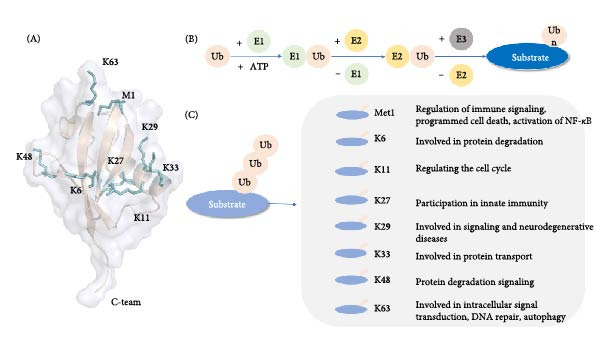
(A) Ubiquitin: basic structure of ubiquitin molecule. (B) Ubiquitination process: ubiquitin ubiquitination of substrate proteins through a three‐level enzyme coupling reaction. (C) Ubiquitin chain function: role of different ubiquitin chains in cell physiological activities.

Ub is a highly conserved small molecule protein composed of 76 amino acids with a molecular weight of ~8.5 kDa (Figure 1). It is widely found in eukaryotes, in the cytoplasm, nucleus, microvilli, autophagic vacuoles, and lysosomes, and is involved in intracellular signal transduction [10]. The most common ubiquitination sites include the N‐terminal methionine site (M1) and seven lysine residues (K6, K11, K27, K29, K33, K48, K63) [2, 11]. Lysine residues can act as Ub‐like receptors to bind to multiple Ub molecules, undergo ubiquitination, and form Ub chains. Proteins are able to bind to a single Ub or Ub polymer on one lysine residue or multiple lysine residues, undergoing monoubiquitination, polyubiquitination, forming monoubiquitin chains, polyubiquitin chains [12, 13]. At present, the most widely studied are the K48 and K63 Ub chains. K48 ubiquitination mediates protein degradation, and K63 ubiquitination mediates signal transduction. It has also been found that the K48‐K63 branch chain can regulate the transduction of nuclear factor kappa B (NF‐*κ*B) [14]. Different Ub chains have different functions (Figure 1). In addition, Ub Lys residues can also be modified by Ub‐like molecules such as small Ub‐like modifier (SUMO) or neural precursor cell expressed developmentally downregulated 8 (NEDD 8). Not only that, Ub can also acetylate at residues other than K29 or phosphorylated at Ser, Thr, or Tyr residues, and each modification has the potential to alter signaling [15].

Ub binds to the substrate by linking the C‐terminal glycine residue to the substrate on the *ε*‐amino group of the substrate lysine residue to make the substrate ubiquitinate, mainly through the continuous action of E1 Ub‐activating enzyme, E2 Ub‐conjugating enzyme, and E3 Ub ligase [15]. The ubiquitination three‐stage enzyme reaction consists of three steps (Figure 1); first, E1 catalyzes the formation of a covalent thioester bond between the cysteine and Ub C‐terminal glycine motifs through magnesium ions and ATP, activates Ub, and second, transfers Ub from E1 to E2 Ub‐binding enzyme [16, 17], The E2–Ub thioester complex (E2‐Ub) is formed, and subsequently the E2‐bound Ub is linked to the target protein by the action of E3 Ub ligase [3, 18]. The specific binding of E3 to the target protein determines the specificity of the ubiquitination process. There are three main types of E3 Ub ligases: E3s with HECT domain, E3s with RING domain, and RING‐between‐RING (RBR) E3s [19–21].

In eukaryotic cells, 80%–90% of protein degradation is done by the UPS, whose main function is to prevent the accumulation of damaged, misfolded, and mutant proteins through proteolysis. E3 Ub ligase specifically recognizes substrate proteins to form target proteins with polyubiquitin chains, which are further recognized and degraded by the 26S proteasome [22]. This degradation process is the use of UPS in living organisms. The 26S proteasome, the main protease in eukaryotic cells, is a large multimeric protease complex responsible for protein degradation in the cytosol and nucleus and is the main component of UPS degradation, which can be divided into two subcomplexes: 19S regulatory particles and 20S proteasome [23]. Many Ub receptors are included in the proteasome, including intrinsic Ub receptors and extrinsic Ub receptors such as regulatory particle non‐ATPase 10 (Rpn10), regulatory particle non‐ATPase 13 (Rpn13), regulatory particle triple‐A ATPase 5 (Rpt5) [24]. Ability to target binding proteins and deliver substrates for degradation [25]. The transcription factor nuclear respiratory factor 1 (Nrf1) is regulated by the proteasome, showing dual roles in the process of UPS; under normal circumstances, Nrf1 is secreted from the endoplasmic reticulum to the cytosol and is degraded by the proteasome after ubiquitination, but when the proteasome is over‐accumulated, the activity is inhibited, or the activity needs to be enhanced, Nrf1 can also be activated by the rapamycin complex (mechanistic target of rapamycin kinase [mTOR]1), indirectly involved in the autophagic process, to promote the metabolic process [26].

### 2.2. Selective Autophagy and ALP

Autophagy plays an important role in the removal of misfolded proteins, damaged organelles, and pathogen removal [6]. Depending on the nutritional profile, autophagy can be divided into selective autophagy and nonselective autophagy [27]. The autophagy process is also known as the ALP, in which autophagic vesicles bind to lysosomes, hydrolyzed proteases hydrolyze phagosomes, and the degraded products are recycled by the body. When the cell is stimulated, selective autophagy is activated, and under certain conditions, autophagosomes are able to specifically isolate and degrade mitochondria, cytoplasmic aggregates, peroxidases, invading pathogens, endoplasmic reticulum, ribosomes, endosomes, lysosomes, lipid droplets, secretory granules [28].

**Figure 2:**
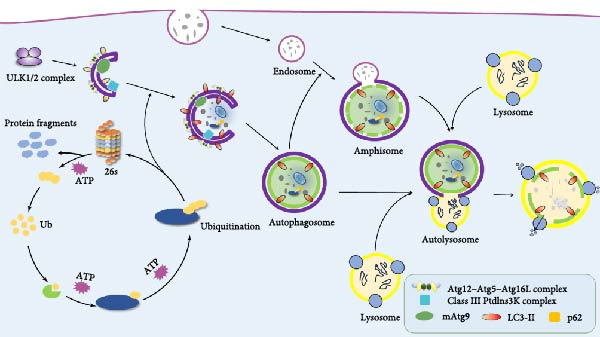
The ubiquitin‐proteasome system and the autophagolysosomal pathway work together to remove abnormal proteins to maintain cellular homeostasis.

Selective autophagy is the selective degradation of specific cellular components by autophagosomes through autophagy; according to this characteristic, selective autophagy can occur both in macroautophagy and in microautophagy and can be divided into mitochondrial autophagy, endoplasmic reticulum autophagy, ribosomal autophagy, and peroxisome autophagy according to the selectivity of autophagy to degrade substrates [29]. The process of autophagy is divided into initiation, nucleation, maturation, and degradation processes. The unc‐51‐like kinase 1 (ULK1) complex is activated by the rapamycin complex (mechanistic target of rapamycin complex [mTORC]1) and adenosine monophosphate (AMP)‐activated protein kinase (AMPK), and autophagy begins [30]. The membrane then swells and nucleates into the nucleation stage, forming a bilayer of separator membrane and phagocytic carrier. This process is mainly mediated by a class III phosphatidylinositol 3‐kinase (PI3K) complex consisting of vacuolar protein sorting 34 (Vps34), vacuolar protein sorting 15 (Vps15), and beclin1 (BECN1). The ULK1 complex phosphorylates and activates the BECN1/Vps34 complex, which is activated to produce phosphatidylinositol‐3‐phosphate (PtdIns3P) on the inner membrane to promote the elongation of autophagic vesicles [31]. The extension and closure of autophagosomes require the involvement of multiple autophagy genes (ATGs), and two Ub‐like coupling systems are required in this process: the ATG5‐ATG12 Ub‐like protein‐coupled system and the ATG8‐phosphatidylethanolamine system [32]. ATG12 and ATG5 are covalently coupled to form the ATG5–ATG12 complex and bind to ATG16 to form a complex to form the E3 ligase of the microtubule‐associated protein microtubule‐associated protein light chain 3 (LC3), which covers the membrane surface and tip and promotes its elongation. When autophagy is induced, LC3 is cleaved by ATG4 proteolysis to produce LC3‐I, and then the carboxy‐terminal glycine exposed by Atg4‐dependent cleavage is activated by E1‐like Atg7 in an ATP‐dependent manner, transferring the activated LC3‐I to ATG3, and then phosphatidylethanolamine is coupled to carboxyglycine to form LC3‐II, which incorporates itself into the autophagosome membrane to drive the extension and closure of the membrane to form autophagosomes [6, 32]. During the maturation stage, autophagosomes bind to lysosomes, and ATG is sequentially removed from the outer membrane of autophagosomes, in which syntaxin 17 (STX17) binds to synaptosomal‐associated protein 29 (SNAP29) and vesicle‐associated membrane protein 8 (VAMP8) to form SNARE complexes and promote their fusion [33]. Subsequently degraded by cells (Figure 2).

Ub ubiquitinates the target protein through a three‐stage enzyme coupling reaction and then uses the 26S proteasome to specifically recognize the target protein with a Ub tag and degrade it into smaller polypeptides, amino acids, and reusable Ub. In addition, the target protein can also recruit selective autophagy adaptor proteins, such as p62, optineurin (OPTN), NBR1 autophagy cargo receptor (NBR1), etc., to bind to Ub through its Ub‐binding domain (UBD) and to participate in the autophagy process through its LC3 interaction region (LIR) motif binding to LC3.

## 3. Ubiquitination Modification Mediates Selective Autophagy

### 3.1. Ub Chains in Cells Mediate Selective Autophagy

Polyubiquitin chains are usually involved in the process of autophagy [13]. Polyubiquitination of K63 chain linkage may be associated with selective autophagy, and the substrates linked to it are able to be recognized by p62/sequestosome 1 (SQSTM1), calcium binding, and coiled‐coil domain 2 (NDP52), OPTN, and NBR1 autophagy receptors. K63 ubiquitination mostly activates or enhances autophagy, while K48‐linked polyubiquitination often leads to proteasomal degradation of signaling molecules, thereby inhibiting autophagy [34]. Saeed has shown that the K63‐Ub chain can be involved in selective autophagy by binding to NBR1 to maintain protein homeostasis [35]. K63‐linked polyubiquitin may bind to p62 to promote autophagy to clear protein contents, and it was further found that lysosomes can efficiently recruit into K63 polyubiquitinated proteins and initiate autophagy [35]. The *Bartonella* T4SS effector BepE can be induced by the K63 chain to select autophagy, and the p62 protein colocalizes with the BepE‐induced autophagosome and targets the degradation of BepE [36]. These all indicate that the K63 chain is involved in selective autophagy.

### 3.2. Bacterial Proteins Mediate Selective Autophagy

Bacterial invasion of the host can cause an immune response, and pathogenic bacteria can produce effector proteins that affect the process of host cells, which is conducive to the survival of pathogens and interferes with the metabolic activities of host cells. In the long‐term coevolution of host and pathogenic microorganisms, pathogenic microorganisms can use the host ubiquitination pathway to promote infection, and many pathogenic bacteria have been found to encode deubiquitinating enzymes to interfere with the host’s ubiquitination system [37].

Selective autophagy is highly dependent on Ub and Ub coupling processes, and bacterial surface proteins or damaged membrane proteins can be directly ubiquitinated, which can recruit related Ub‐binding autophagy receptors, such as p62/SQSTM1, NBR1, NDP52, tax1 binding protein 1 (TAX1BP1), and OPTN [38, 39]. They have the same/similar domain, with a UBD/Ub‐binding zinc finger domain (ZZ) and LIR motif, capable of binding to Ub, LC3 [39]. It is able to recognize Ub proteins on bacterial surfaces and link ubiquitination targets to autophagy mechanisms for autophagic lysosomal degradation. The formation of autophagosomes is the manifestation of cells fighting bacterial infection, and the autophagic proteins involved in this process mainly include ULK1, ATG5, ATG16L1, and BECN1; in addition, intracellular bacteria can interfere with the autophagy process for escape and replication survival. Moreover, the bacteria‐specific secretion system is able to inhibit autophagosome formation [40]. Many bacteria are able to manipulate ubiquitination during autophagy and even prevent the maturation of autophagosomes, thus evading autophagy‐dependent degradation [41]. The host is able to remove foreign microorganisms using UPS and ALP, and inhibition of UPS and ALP significantly reduces the survival of *Brucella* in RAW264.7 cells [42, 43]. The PE/PPE protein family in *Mycobacterium tuberculosis* (Mtb) can use hydrophobic interaction to bind to free Ub, promote Ub‐binding autophagy receptor recognition, participate in autophagy, induce the clearance of specific proteins, and maintain long‐term survival by reducing bacterial inflammation. In addition, PE protein can regulate mTOR to inhibit autophagy, thereby evading host immunity and autophagy clearance, thereby achieving survival [44]. The *Salmonella* effector SopA is a HECT‐like E3 Ub ligase that is ubiquitinated by the host E3 enzyme HsRMA1, and SopA ubiquitination helps *Salmonella* escape from *Salmonella*‐containing vacuoles (SCVs) and avoid clearance [45].

### 3.3. Viral Proteins Mediate Selective Autophagy

Viral infection can lead to a variety of physiological changes in host cells and affect physiological functions. Viral infection activates autophagy, which protects cells by restricting viral replication, but autophagy plays an active role in viral replication, and viruses use the process of autophagy to enhance their own replication and survival [46].

Similar to bacterial infection, the body can target the degradation of viral components and viral particles through selective autophagy, in which the ubiquitination of viral proteins can be recognized by the autophagic cargo receptors NDP52, p62/SQSTM1, etc., and chelated into lysosomes in an LC3‐dependent manner, which are degraded by hydrolase enzymes. In addition, viruses are able to use the host’s Ub mechanism to inhibit autophagy to promote their own replication and survival and can also disrupt the host Ub mechanism to resist innate immune responses [47]. p62/ SQSTM1 is the first selective autophagy adaptor protein to be discovered, which is a multifunctional protein that plays an important role in clearing the protein to be degraded and maintaining intracellular protein homeostasis [48]. p62 is phosphorylated at S403 and is a key component of the autophagic degradation of polyubiquitinated proteins [49]. p62 can target the viral capsid for autophagic degradation and exhibit antiviral effects [46]. p62‐mediated selective autophagy targets viral proteins 1 (VP1) and viral proteins 3 (VP3) for degradation to inhibit Seneca virus (SV) replication [50]. Wang et al. [51] found that p62‐mediated selective autophagy can be spontaneously induced and participate in the reactive oxygen species (ROS)‐Keap1‐Nrf2 pathway, and inhibition of autophagy can promote p62 accumulation and nuclear translocation to exacerbate ROS‐induced DNA damage, further supporting the DNA damage response of p62‐mediated autophagy to promote Epstein–Barr virus (EBV) latency. It is also able to interfere with the cell cycle and enhance self‐replication by selecting host ubiquitinated proteins [51]. S‐phase kinase‐associated protein 2 (SKP2) is an E3 ligase that can reduce BECN1 ubiquitination, reduce BECN1 degradation and enhance autophagy, which can lead to a decrease in BECN1 and block autophagy during replication, and inhibition of SKP2 can enhance autophagy and reduce Middle East respiratory syndrome coronavirus (MERS‐CoV) replication [52]. At the same time, viral proteins can be directly modified by Ub, which is significantly involved in the process of virus budding and release [53]. Gag protein ubiquitination of human immunodeficiency virus type 1 (HIV‐1) has been shown to be related to the ability of viral particles to release viral particles, coordinating the assembly of retroviral particles by providing viral particle components that recruit cellular cofactors [54]. Virus‐infected host cells have an innate antiviral response that induces an intracellular signaling cascade to maximize response to infection. Many proteins are involved in the regulation of antiviral signaling pathways, including ubiquitinase.

**Figure 3:**
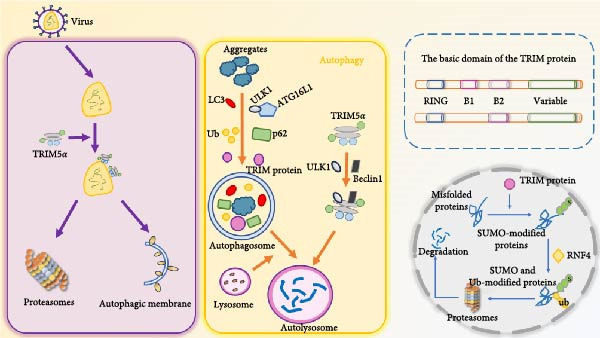
The TRIM protein is involved in autophagy and the ubiquitin protein system to exert antiviral effects. TRIM, tripartite motif.

Tripartite motif (TRIM) protein is a multifunctional family of Ub E3 ligases, which are involved in intracellular signal transduction, innate immunity, autophagy, transcription, and other physiological activities as E3 Ub ligases, and play an important role in the process of antiviral infection [55, 56] (Figure 3). TRIM protein can participate in autophagy‐mediated antiviral defense processes and regulate innate immune processes to maintain antiviral function by directly targeting viral components [57]. TRIM5*α* limits infection by directly interacting with the HIV‐1 viral capsid and induces premature undressing and capsid breakdown; TRIM5*α* directly recognizes its homologous retroviral capsid and coordinates its autophagic degradation without Ub labeling, thereby protecting cells from HIV‐1 infection [56]. TRIM23 can form complexes with TANK‐binding kinase 1 (TBK1) and p62 to promote TBK1‐mediated phosphorylation of p62, thereby promoting autophagy [58].

The basic domains of TRIM proteins include the N‐terminal ring finger domain, the ZZ of one or two B‐boxes (B1 box and B2 box) and their associated coiled helix regions, and the C‐terminal structure is variable due to its main involvement in substrate recognition, mainly including COS domain, fibronectin type III repeat (FNIII), PRY domain, SPRY domain, etc. TRIM protein can play an antiviral role by directly targeting viral components, regulating autophagy, and mediating antiviral immunity. TRIM5*α* restricts retroviral infection by directly interacting with the viral capsid and inducing premature uncoating and capsid breakdown, in addition to being able to participate in the autophagy process, and the TRIM protein is able to act as an E3 ligase targeting substrate for protein ubiquitination.

## 4. Ubiquitination and Selective Autophagy in Zoonotic Diseases

### 4.1. Salmonellosis

**Figure 4:**
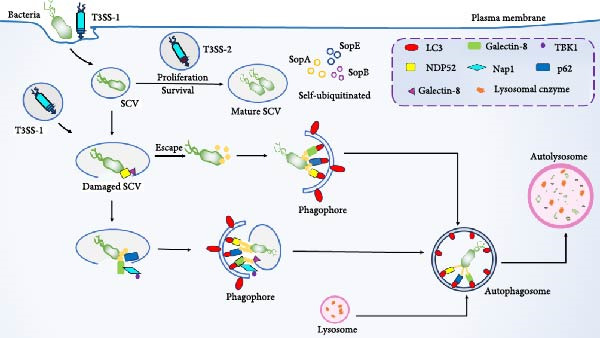
Ubiquitination and autophagy after *Salmonella* infection.

After *Salmonella* invades the host cell, it is encapsulated by the phagocytic membrane to form an SCV, and Ub molecules or Ub‐binding proteins can accumulate on the surface of the SCV membrane, causing it to undergo ubiquitination, which is further recognized by selective autophagy receptors, and then cleared by selective autophagy (Figure 4) [59]. E3 Ub ligase can inhibit the intracellular proliferation of *Salmonella* and can not cause the production of bacterial Ub shell in cells infected with *Salmonella* knockout E3 Ub ligase ring finger protein 213 (RNF213), thereby inhibiting the recruitment of NDP52 and p62, thereby reducing the occurrence of autophagy much so that *Salmonella* proliferation was not inhibited in RNF213‐deficient cells [60].

Host cell‐mediated *salmonella* ubiquitination can be used as a signal transduction platform for cellular defense mechanisms, and *Salmonella* effector proteins can regulate the Ub system of host cells, which further affects the self‐clearance of selective autophagy (Figure 4). SCV maturation depends on the type 3 secretion system (T3SS) encoded by *Salmonella* pathogenicity island 1 (SPI‐1) and *Salmonella* pathogenicity island 2 (SPI‐2); SPI‐1‐encoded T3SS can mediate bacterial invasion of the host, SPI‐2‐encoded T3SS is involved in bacterial replication and survival, and *Salmonella* can use the T3SS effector system to block the ubiquitination process, evade autophagic clearance, and increase survival efficiency [59, 61]. *Salmonella* is able to secrete effector proteins, such as SopA, SopB, SopE, etc., which affect the antimicrobial response through self‐ubiquitination [62]. SopA can directly mediate the ubiquitination and proteasomal degradation of TRIM56 and TRIM65, and the catalytic activity of SopA enhances the expression of interferon *β* (IFN‐*β*) stimulated by TRIM56 and TRIM65 and regulates innate immunity [63, 64]. SopB mediates vimentin IF aggregation and further promotes the replication of *Salmonella* in SCV [65]. In addition, SopB also inhibits autophagy by activating the mTORC1 complex, increasing the survival of *Salmonella* in B cells [66]. SopE is able to directly bind to the host transcription factor SP1 to regulate ATG LC3B expression, and only when *Salmonella* is present in SCV, SopE regulates autophagic flux [67]. SPI‐2 can further disrupt autophagy by damaging the mTOR signaling pathway to facilitate its own survival [68]. *Salmonella* secreted effector L (SseL) is an effector protein of *Salmonella*, which can act as a deubiquitinating enzyme to inhibit the recognition of Ub aggregates by autophagy, reduce the Ub levels of p62 and LC3 in SCV‐related aggregates, interfere with the ubiquitination pathway, reduce autophagic flux, and promote survival [45, 59]. This further indicates that SseL reduces the occurrence of autophagy and maintains survival by disrupting ubiquitination [62, 69]. It has also been reported that *Salmonella* typhimurium infects the host in a type I IFN (IFN‐I) dependent manner, attenuates the lysosomal degradation of TRIM21 through chaperone‐mediated self (CMA), and induces mTORC2/Akt signaling to enhance the expression of TRIM21 [70].

After *Salmonella* invades the host cell, it is encapsulated by the phagocytic membrane to form SCVs, which can undergo ubiquitination and be recognized by selective autophagy receptors. SCV can protect *Salmonella* from being destroyed by T3SS‐1, and replicate and survive under the action of T3SS‐2 in SCV. However, after the destruction of T3SS‐1, the SCV ruptures, and a part of *Salmonella* escapes into the cytoplasm, which can be quickly recognized and ubiquitinated by Ub and selectively recruited by autophagy receptors (p62, NBR1, NDP52, etc.), and degraded by autophagy. In addition, *Salmonella* effector proteins SopA, SopB, and SopE can self‐ubiquitinate to affect antimicrobial responses.

### 4.2. Brucellosis

*Brucella* is capable of invading and replicating monocytes and macrophages, causing persistent infection of the host. Much evidence suggests that the host innate immune response plays a key role in *Brucella* infection. *Brucella* invades host cells and forms *Brucella*‐containing vacuoles (BCVs) in the membrane‐bound diaphragm and is covered by endoplasmic reticulum‐derived replicating organelles under the control of *Brucella*, further restricting BCV and lysosomal fusion to avoid degradation [71]. In the early stage of *Brucella* infection, the formation of replicating BCV (rBCV) originating from the endoplasmic reticulum requires the participation of autophagy‐related proteins ATG9 and WD‐repeat protein interacting with phosphoinositides (WIPIs), and rBCV requires the participation of autophagy initiation proteins BECN1, PI3K, ULK1, and Atg14L to be converted into autophagic BCV (aBCV), and the formation of aBCV accelerates the process of intracellular circulation of *Brucella* [72, 73]. In addition, the type IV secretion system of *Brucella* is also involved in the regulation of this process. Jing found that 2308ΔVceA promoted autophagy and inhibited apoptosis in HPT‐8 cells during *Brucella* abortus infection after constructing a VceA mutant strain (2308ΔVceA) of *Brucella* abortus 2308 (S2308) and infecting human trophoblast cells (HPT‐8 cells) [74]. *B. neotomae* induces phosphorylation and autophagy of p38 mitogen‐activated protein kinase in a type IV secretion system‐dependent manner, and the intersection of the MAP kinase pathway and autophagy machinery is a key component of the intracellular life cycle of *Brucella* [75].

Host protein ubiquitination also plays an important role in *Brucella* infection of macrophages. *Brucella* infection can induce the increase of E3 Ub ligase neuronal precursor cell‐expressed developmentally downregulated 4 (NEDD4) activity in a calcium‐dependent manner, and NEDD4 ubiquitination can lead to the degradation of calpain 2 induced by *Brucella* infection, thereby inhibiting macrophage apoptosis [76]. Kiranmai elucidated that the host Ub‐specific protease USP8 negatively regulates *Brucella* invasion into macrophages via the plasma membrane receptor CXCR4 [77]. However, the molecular mechanisms by which *Brucella* and related virulence factors affect host Ub modification and immune response are not well understood. *B. melitensis* UTP‐glucose‐1‐phosphotransferase (UGPase) inhibits the activation of NF‐*κ*B by regulating the ubiquitination levels of K63 (K63‐Ub) and Met1 (Met1‐Ub) in NEMO, thereby avoiding host immune responses and enhancing intracellular survival [78]. Although the specific mechanism of *Brucella* demediating autophagy through protein ubiquitination modification is not clear, it is still experimental to prove that inhibition of UPS can activate ALP after infection of RAW264.7 cells by *Brucella* porcine, but inhibition of ALP can effectively activate UPS, and when UPS and ALP are inhibited at the same time, it can fully and effectively inhibit the survival of *Brucella* in cells [42].

### 4.3. Tuberculosis

Mtb is able to be engulfed by macrophages upon invasion, but Mtb utilizes a variety of strategies to avoid or inhibit host defense mechanisms, inhibiting the innate immune response by modulating host‐critical signaling pathways and altering posttranslational modifications, thereby favorably establishing infection for survival [44, 79, 80]. Eukaryotic‐type protein kinase G (PknG) enhances the ubiquitination and degradation of tumor necrosis factor (TNF) receptor‐related factor 2 (TRAF2) and transforming growth factor‐*β* (TGF‐*β*)‐activated kinase 1 (TAK1), thereby inhibiting NF‐*κ*B signaling and activation of host innate responses [81]. Mtb can use host autophagy and Ub mechanism to resist immune response, host E3 ligases Pyk2‐related nonkinase (PRNK) and Smurf1 can resist Mtb infection and establish immune defense, Ub binds to Mtb‐containing vacuoles to recruit autophagic linkers, such as p62, NDP52, NBR1, OPDN, etc., to couple LC3‐II. Into ubiquitinated Mtb vacuoles and deliver them to autolysosomes for targeted degradation [44, 82]. Ub can recognize the Mtb surface protein Rv1468c, which contains a UBD domain that can directly bind Ub to recruit p62 and deliver Mtb to autophagosomes containing LC3, and the number of Mtb in autophagic vesicles decreased significantly after knocking out Rv1468c, indicating that the host can use Rv1468c to clear Mtb through macroautophagy [83].

In addition, host intracellular E3 ligase can target autophagy to affect Mtb survival in the cell. TRIM32 promotes xenoggy by promoting BECLIN1 recruitment of Mtb to form autophagosomes and ubiquitination‐mediated autophagocytosis of Mtb to promote Mtb clearance [84]. Smurf1 is involved in polyubiquitin recruitment and bacterial autophagy and is able to comediate Ub‐dependent selective autophagy of Mtb with Parkin, inhibiting the replication of Mtb in mouse macrophages, in which Smurf1 mediates K48‐linked ubiquitination of Mtb by relying on its Ub ligase activity and C2 domain. Tyrosine phosphatase protein tyrosine phosphatase A (PtpA), a secreted effector protein required for survival within Mtb cells, suppresses innate immunity by selecting the host Ub system (85), PtpA inhibits innate immunity to the kinases JNK and p38 and the transcription factor NF‐*κ*B pathway by competitively binding to the UBD domain of the host adaptor protein TAB3 [85], TRIM27 is identified as a host protective factor targeting Mtb, inhibiting Mtb survival by enhancing the JNK/p38 pathway‐mediated host immune inflammatory response as well as apoptosis, and PtpA can antagonize TRIM27‐promoted JNK/p38 MAPK pathway activation and apoptosis by competitively binding to the RING domain of TRIM27 [86]. TRIM27 enhances host protective immunity by activating transcription factor EB (TFEB), which binds to the TFEB promoter and the TFEB transcription factor cAMP response element‐binding protein 1 (CREB1), thereby enhancing CREB1‐TFEB promoter binding affinity and promoting the transcriptional activity of CREB1 against TFEB, ultimately inducing autophagy‐related gene expression as well as autophagic flux activation to clear pathogens [87].

### 4.4. Rabies

Rabies virus (RABV) can cause autophagic vesicle accumulation during infection and can increase autophagic flux by increasing the rate of autophagosome synthesis and decreasing the rate of degradation or decrease autophagic flux by decreasing the rate of lysosomal degradation to maintain autophagic replication and survival [88]. RABV phosphoprotein (RABV‐P) plays an important role in viral replication, and RABV‐P has been found to bind to TBK1 and interfere with the formation of innate immune condensates [89]. RABV‐P P5 can bind to the circular structure of BECN1 and induce incomplete autophagy through the BECN1‐mediated signaling pathway, thereby promoting RABV replication [90]. IFN‐induced transmembrane protein 3 (IFITM3) was identified as an important antiviral host effector for IFN‐I induction, demonstrating that IFITM3 is a key inhibitor of RABV replication and also inhibits viral replication through mTORC1‐dependent autophagy [91].

TRIM protein plays a role in virus‐mediated autophagy. Zhang et al. [92] found that the expression of TRIM21 was upregulated in the brain tissue of RABV‐infected mice, and TRIM21 knockdown could inhibit RABV replication and further found that TRIM21 ubiquitination degraded IFN regulatory factor 7 (IRF7) to regulate RABV replication [92]. He et al. [93] also found that TRIM44 protein also has a similar effect in RABV infection; TRIM44 can activate autophagy and promote viral replication, silencing TRIM44 inhibits RABV replication, and TRIM44 overexpression promotes RABV replication [93]. Another study found that TRIM72 overexpression significantly reduced the viral titer of RABV in neuronal cells and alleviated the pathogenicity of RABV in mice. It also promotes the ubiquitination of RABV matrix protein (RABV‐M) K48 linkage so that M can be degraded through the proteasomal pathway [94].

The Pro–Pro‐x (any amino acid)‐Tyr (PPxY) motif can recruit the E3 ligase NEDD4 and bind to the WW domain and the Ub protein, which plays an important role in the process of virus budding; the PPxY motif is conserved in RABV, and the Ub ligase Rsp5 can interact with the RABV‐M in a PY‐dependent manner [95–97]. Free Ub is essential for viral budding, and proteasome inhibitors (MG132) can block viral release and reduce viral budding efficiency [97]. The PPxY motif of the RABV‐M protein interacts with the E3 Ub‐protein ligase NEDD4. Upon binding to ubiquitinated RABV‐M, NEDD4 can bind more LC3 and enhance autophagosome accumulation, while NEDD4 knockdown significantly reduces M‐induced autophagosome accumulation. Further have shown that RABV‐M prevents autophagosome‐lysosomal fusion and promotes viral budding. Inhibition of RABV‐M‐induced autophagosome accumulation reduces the production of extracellular virus‐like particles [98].

### 4.5. Toxoplasmosis

*Toxoplasma gondii* invades the host through an invasion mechanism and uses the host cell membrane to encapsulate itself into parasitic vacuoles (PVs) into the cell [99, 100]. PV can protect *T. gondii*, reduce host immune defense, enhance replication ability, and increase intracellular survival time [101]. During *T. gondii* infection, autophagy can be stimulated by CD40 (CD40 molecule) and autophagy targeting, while blocking the signaling pathways epidermal growth factor receptor (EGFR), PI3K, or Akt leads to autophagy targeting of PV and parasite killing [101, 102].

**Figure 5:**
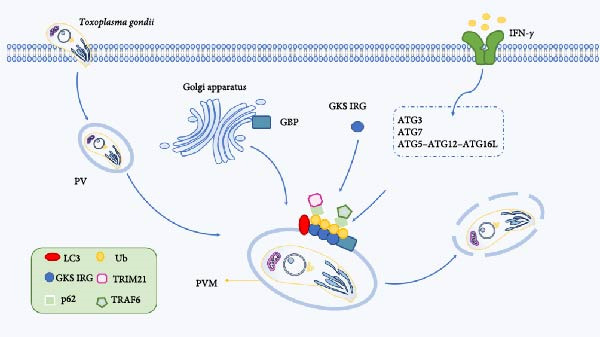
IFN‐*γ* has a variety of anti‐*Toxoplasma gondii* activities against infected cells.

Ub‐like/Ub‐associated proteins (UBL‐UBA) shuttle protein plays a key role in regulating the degradation and cell division of *T. gondii* ubiquitinated protein. Shuttle protein is localized in both the cytoplasm and nucleus of *T. gondii*, and the deletion of shuttle protein can inhibit the growth of *T. gondii* and the accumulation of Ub protein [103]. The results showed that the ubiquitination protein was upregulated after *T. gondii* infection, and the level of ubiquitination was related to the cell cycle process [104]. IFN‐*γ* is a key factor in the immune response of cells to pathogens, and after *T. gondii* invades the host, the level of IFN‐*γ* increases, Ub, p62, NDP52, and LC3 are recruited to the parasitic vacuolar membrane (PVM), and the parasite is encapsulated by multimembrane structure, resulting in growth retardation [105]. A similar mechanism was found in IFN‐*γ*‐induced cells, where PV was able to be labeled by Ub molecules and further targeted by autophagy to impairing intracellular parasite replication, and p62 was recruited into Toxoplasma‐containing PV in an IFN‐*γ*‐dependent manner [106]. Autophagic proteins function by promoting the recruitment of immunomodulatory GTPases (immunity‐related GTPases [IRGs]) and guanylate binding proteins (GBPs) to PVMs, and IFN‐*γ* induces the recruitment of the GKS subfamily of IRG effectors to PVMs, resulting in membrane structural disruption, which enables GBPs to bind to and kill parasites [101]. To further determine PV degradation, Choi et al. [107] demonstrated that the Ub‐like coupling mechanisms of the autophagy pathways, E1 Atg7, E2 Atg3, and E3 Atg12‐Atg5‐Atg16L1, are required for proper targeting of IFN‐*γ* effectors to the PVM of *T. gondii* and subsequent control of *T. gondii* infection in vitro and in vivo (Figure 5) [107].

Immune‐related p47GTPases (IRGs) and p65 GTPases (GBPs) located in the Golgi apparatus and endoplasmic reticulum are recruited to the PVM and induce subsequent autophagic molecules to destroy the parasite vacuoles. IFN‐*γ* can use the Ub‐like coupling mechanism E1 Atg7, E2 Atg3, and E3 Atg12‐Atg5‐Atg16L1 to recruit GKS IRG and GBP to correctly target IFN‐*γ* effectors to the PVM of *T. gondii* for targeted degradation.

The body can use UPS and ALP to eliminate *T. gondii* in the body and enhance the body’s immunity, but on the contrary, *T. gondii* can also hijack Ub or interfere with the Ub system and autophagy pathway to promote its own survival. TRIM21 protein transcription and translation levels are upregulated after *T. gondii* infects cells, and TRIM21 knockout mice are highly sensitive to *T. gondii*, with increased parasites in the peritoneum and brain, and IFN‐*γ* induces TRIM21 recruitment to *T. gondii* vacuoles, resulting in Lys63‐linked ubiquitination of vacuoles and inhibition of early replication of *T. gondii* [108]. Further have found that the *T. gondii* virulence factor ROP18 can interact with TRIM21 protein, promote the phosphorylation of the latter, and promote the degradation of TRIM21 through the lysosomal pathway, leading to immune escape [109]. In the process of *T. gondii* infection of mouse intestinal cells, it was found that the secretion of *T. gondii* rhoptry organelle protein 17 (ROP17) by *T. gondii* was activated by BECN1‐bcl‐2‐dependent autophagy, which was conducive to the survival and proliferation of the intracellular protist *T. gondii* [110]. In addition, *T. gondii* can also inhibit the transcriptional program regulated by Forkhead box O3 (FOXO3*α*), which further inhibits the autophagy killing of host cells [111]. Ovarian tumor domain (OTU)‐containing deubiquitinase (DUB) also plays a role in *T. gondii* growth, with a significant reduction in intracellular plaque size after knockdown of TgOTUD6B, in addition to the preferential targeting of K6, K11, K48, and K63‐linked ubiquitinated substrates to promote degradation [112].

## 5. Conclusion

The two major pathways of protein degradation in cells are the UPS and the ALP, and the main function of UPS is to prevent the accumulation of damaged, misfolded, and mutant proteins through proteolysis. ALP can form autophagosomes during autophagy, wrap the substances to be degraded, and then combine with lysosomes to form autophagic lysosomes, which are degraded into small molecules by hydrolytic proteases, which are reabsorbed and utilized by the body. Since the Ub‐like protein‐coupled system is involved in the autophagy process, UPS and ALP can be linked, and not only that, both UPS and ALP are sufficient to degrade ubiquitinated substrates and ubiquitinated proteasomes can be cleared by the autophagic pathway, and autophagy‐related proteins can also be degraded and cleared by the proteasome pathway.

In the process of symbiosis between bacteria, viruses, and parasites, they can use the host ubiquitination pathway and autophagy pathway to promote infection and enhance their replication and survival in host cells. Direct ubiquitination of surface proteins of pathogenic microorganisms recruits selective autophagy receptors, interferes with the autophagy process, maintains self‐survival and resists host immune defense, and can also mimic host proteins to form new effectors to interfere with host Ub pathways. Ub E3 ligase is able to specifically recognize substrates, while deubiquitinating enzymes counteract the ubiquitination activity of E3 ligase by selectively removing Ub chains from target proteins. Pathogenic microorganisms can use this mechanism to escape and increase the chance of survival. Since pathogenic microorganisms use UPS and ALP to promote their own replication and survival, while proteasome inhibitors and autophagy inhibitors can inhibit protein activity, thereby reducing protein degradation and protecting important proteins in cells from degradation, inhibitors can be used to block the ubiquitination process and autophagy process, and then the survival of pathogenic microorganisms in the cell can be explored from the inhibitor aspect, which provides a new strategy for the body to improve immune defense.

**Nomenclature**
UPS: Ubiquitin‐proteasome system
ALP: Autophagic lysosomal pathway
SUMO: Small ubiquitin‐like modifier
NEDD 8: Neural precursor cell expressed developmentally downregulated 8
RBR: RING‐between‐RING
Rpn10: Regulatory particle non‐ATPase 10
Rpn13: Regulatory particle non‐ATPase 13
Rpt5: Regulatory particle triple‐A ATPase 5
Nrf1: Nuclear respiratory factor 1
mTOR: Mechanistic target of rapamycin kinase
mTORC1: Mechanistic target of rapamycin complex 1
ULK1: Unc‐51‐like kinase 1
AMP: Adenosine monophosphate
STX17: Syntaxin 17
SNAP29: Synaptosomal‐associated protein 29
VAMP8: Vesicle‐associated membrane protein 8
NF‐*κ*B: Nuclear factor kappa B
AMPK: AMP‐activated protein kinase
PI3K: Phosphatidylinositol 3‐kinase
Vps34: Vacuolar protein sorting 34
Vps15: Vacuolar protein sorting 15
BECN1: Beclin1
PtdIns3P: Phosphatidylinositol‐3‐phosphate
ATG: Autophagy gene
LC3: Microtubule‐associated protein light chain 3
p62/SQSTM1: sequestosome 1
OPTN: Optineurin
NBR1: NBR1 autophagy cargo receptor
NDP52: Calcium binding and coiled‐coil domain 2
TAX1BP1: Tax1 binding protein 1
UBD: Ubiquitin‐binding domain
ZZ: Zinc finger domain
LIR: LC3 interaction region
fPE/PPE: Proline‐glutamate/proline–proline‐glutamate
SopA: *Salmonella* secretes proteins SopA
SopB: *Salmonella* secretes proteins SopB
SopE: *Salmonella* secretes proteins SopE
HsRMA1: E3 enzyme HsRMA1
SCVs: *Salmonella*‐containing vacuoles
VP1: Viral proteins 1
VP3: Viral proteins 3
SV: Seneca virus
EBV: Epstein–Barr virus
SKP2: S‐phase kinase‐associated protein 2
MERS‐CoV: Middle East respiratory syndrome coronavirus
TRIM: Tripartite motif
TBK1: TANK‐binding kinase 1
RNF213: E3 ubiquitin ligase ring finger protein 213
T3SS: The type 3 secretion system
SPI‐1: *Salmonella* pathogenicity island 1
SPI‐2: *Salmonella* pathogenicity island 2
IFN‐*β*: Interferon *β*
SseL: Salmonella Secreted Effector L
IFN‐I: Type I interferon
CMA: Chaperone‐mediated self
*Mtb*: *Mycobacteriumtuberculosis*
PknG: Eukaryotic‐type protein kinase G
TRAF2: TNF receptor‐associated factor 2
TAK1: Transforming growth factor‐*β*‐activated kinase 1
PRNK: Pyk2‐related nonkinase
Rv1468c: Mtb surface protein Rv1468c
PtpA: Protein tyrosine phosphatase A
TFEB: Transcription factor EB
CREB1: cAMP response element‐binding protein 1
RABV: Rabies virus
RABV‐P: Rabies virus phosphoprotein
IFITM3: Interferon‐induced transmembrane protein 3
IRF7: Interferon regulatory factor 7
PPxY: Pro–Pro‐x (any amino acid)‐Tyr
NEDD4: Neuronal precursor cell‐expressed developmentally downregulated 4
RABV‐M: Rabies virus matrix protein
PVs: Parasitic vacuoles
CD40: CD40 molecule
EGFR: Epidermal growth factor receptor
Ub: Ubiquitin
UBL‐UBA: Ubiquitin‐like/ubiquitin‐associated proteins
IFN‐*γ*: Interferon‐*γ*
PVM: Parasitic vacuolar membrane
IRGs: Immunity‐related GTPases
GBPs: Guanylate binding proteins
ROP17: *Toxoplasma gondii* rhoptry organelle protein 17
OTU DUB: Ovarian tumor domain‐containing deubiquitinating enzymes
FOXO3*α*: Forkhead box O3.

## Conflicts of Interest

The authors declare no conflicts of interest

## Author Contributions

All authors were involved in the writing and revision of the manuscript content of the paper. Chi Meng, Fengyuan Jiao, Gengxu Zhou, and Zuoyong Zhou contributed equally to this work.

## Funding

This study was financially supported by the State Key Laboratory for Animal Disease Control and Prevention (SKLVEB‐KFKT‐09), the Fundamental Research Funds for the Central Universities (SWU‐KT22013), Science and Technology Major Project of Gansu Province (22ZD6NA001), Innovation Program of Chinese Academy of Agricultural Sciences (CAAS‐CSAB‐202403), and the Natural Science Foundation of Chongqing (2022NSCQ‐MSX2392, cstc2020jcyj‐msxmX0446).

## Data Availability

Data sharing is not applicable to this article as no datasets were generated or analyzed during the current study.
